# Constant luminance (cd·s/m^2^) versus constant retinal illuminance (Td·s) stimulation in flicker ERGs

**DOI:** 10.1007/s10633-017-9572-3

**Published:** 2017-02-03

**Authors:** C. Quentin Davis, Olga Kraszewska, Colette Manning

**Affiliations:** 1 LKC Technologies, Inc., 2 Professional Drive Suite 222, Gaithersburg, MD 20879 USA; 2 Wedgwood Optometry, Fort Worth, TX USA

**Keywords:** Flicker electroretinogram (ERG), Troland, Pupil, Dilation, Reference range, Mydriasis

## Abstract

**Purpose:**

To compare the effect of variable pupil size on the flicker electroretinogram (ERG) between a stimulus having constant luminance and a stimulus having constant retinal illuminance (constant Troland) that compensates for pupil size.

**Methods:**

Subjects (*n* = 18) were tested with 12 pairs of the stimuli. The stimulus pair consisted of the ISCEV standard constant luminance stimulus (3 cd·s/m^2^ with a 30 cd/m^2^ background) and a constant retinal illuminance stimulus (32 Td·s with a 320 Td background) selected to provide the same stimulus and background when the pupil diameter is 3.7 mm. Half the subjects were artificially dilated, and their response was measured before and during the dilation. The natural pupil group was used to assess intra- and inter-subject variability. The artificially dilated group was used to measure the flicker ERG’s dependence on pupil size.

**Results:**

With natural pupils, intra-subject variability was lower with the constant Troland stimulus, while inter-subject variability was similar between stimuli. During pupil dilation, the constant Troland stimulus did not have a dependence on pupil size up to 6.3 mm and had slightly larger amplitudes with longer implicit times for fully dilated pupils. For the constant luminance stimulus, waveform amplitudes varied by 22% per mm change in pupil diameter, or by 48% over the 2.2 mm diameter range measured in dilated pupil size. There was no difference in inter-subject variability between constant Troland natural pupils and the same subjects with a constant luminance stimulus when dilated (i.e., the ISCEV standard condition).

**Conclusions:**

These results suggest that a constant Troland flicker ERG test with natural pupils may be advantageous in clinical testing. Because of its insensitivity to pupil size, constant Troland stimuli should produce smaller reference ranges, which in turn should improve the sensitivity for detection of abnormalities and for monitoring changes. In addition, the test can be administered more efficiently as it does not require artificial dilation.

**Clinical Trial registration number:**

This trial is registered at ClinicalTrials.gov (NCT02466607).

## Introduction

The flicker electroretinogram (ERG) measures the retina’s response to an intermittent flash stimulus, flickering at about 30 Hz [1]. Changes in the flicker ERG are clinically useful in a variety of conditions, such as retinitis pigmentosa [2–5], diabetic retinopathy [6–9], central retinal vein occlusion [10–13], and vigabatrin toxicity testing [14].

According to the ISCEV standard [1], the flicker ERG is performed with dilated pupils and brief full-field flashes of white light with a time-integrated luminance of 3 cd·s/m^2^ on a 30 cd/m^2^ background. The purpose of dilating the pupils is to provide a more consistent pupil size and therefore reduce variations in retinal stimulation created by differing amounts of light entering the eye. The RETeval device (LKC Technologies, Inc., Maryland USA; also sold as RETeval-DR by Welch Allyn Inc., New York, USA) can measure both the ERG and pupil size. The device can dynamically change the stimulus intensity according to the pupil size to deliver a constant retinal illuminance according to the formula retinal illuminance equals the product of the luminance and the pupil area [15]. When the luminance is measured in cd/m^2^ and the pupil area is measured in mm^2^, the retinal illuminance is measured in Trolands, or Td. For brief flashes, the time-integrated retinal illuminance is measured in Troland seconds, or Td·s.

In a recent publication [15], flicker ERGs were recorded using a 8 Td·s stimulus in subjects at 7 time points as they were being artificially dilated. By testing the same subject as they are being dilated, the expectation was that the ERG should remain constant over the dilation period if the 8 Td·s stimulus correctly compensated for dilation by providing a consistent retinal stimulus. Their results found that the amplitude of the flicker ERG was independent of pupil size, and the implicit time was independent for pupil sizes smaller than 6.5 mm. For pupil diameters greater than 6.5 mm, the implicit time was delayed (up to about 1 ms) with respect to the natural pupil condition.

The aim of this work is to extend previous work [15] to compare constant luminance and constant retinal illuminance stimuli in order to determine the pupillary dependence of both methods. A further aim of this work is to estimate the contribution of variable pupil size (in both natural and dilated pupils) on flicker ERG parameters. This work includes the ISCEV standard flicker stimulus with dilated pupils, as well as other stimulus and pupillary states.

## Methods

### Study design

In a single session, one randomly selected eye of each subject was tested every 3 min with a stimulus pair. After the second stimulus pair (3.5 min after the beginning of the test), half the subjects had 0.5% tropicamide instilled into the tested eye to begin the process of artificial dilation (1 drop for subjects with light irides, 2 drops for dark irides). The other half of the subjects were not artificially dilated. Testing with the stimulus pair was repeated every 3 min for a total of 12 times over the course of the 33.5-min procedure.

The stimulus pair consisted of (1) the ISCEV standard constant luminance stimulus (3 cd·s/m^2^ with a 30 cd/m^2^ background) and (2) a constant retinal illuminance stimulus (32 Td·s with a 320 Td background) selected to provide the same stimulus and background when the pupil diameter is 3.7 mm $\left( {3 \frac{{{\text{cd} \cdot \text{s}}}}{{{\text{m}}^{ 2} }} \pi \left( {\frac{3.7}{2}\,{\text{mm}}} \right)^{2} = 32\, {\text{Td}\!\cdot\!\text{s,}}\, \text{and }\, 30 \frac{\text{cd}}{{{\text{m}}^{ 2} }} \pi \left( {\frac{3.7}{2}\,{\text{mm}}} \right)^{2} = 320\, {\text{Td}}} \right)$. The choice of 3.7 mm attempts to minimize the maximum difference in retinal illumination (stimulus and background) between the two stimuli: Compared to the constant Troland stimulus, the retinal illumination for the constant luminance stimulus is $\left( {\frac{{3.7 \, {\text{mm}}}}{{1.8 \, {\text{mm}}}}} \right)^{2} = 4.2 \times$ dimmer for the smallest pupil in the study (Fig. 3) and $\left( {\frac{{8.1 \, {\text{mm}}}}{{3.7 \, {\text{mm}}}}} \right)^{2} = 4.8 \times$ brighter for the largest pupil in the study.

The stimulus pair both used white light (CIE 1931 chromaticity of (0.33, 0.33)) as synthesized from RGB LEDs with a 28.3 Hz flicker frequency [9]. To make flashes of light, the RETeval device varied the flash duration for a 6000 cd/m^2^ luminance; thus, the constant luminance stimulus had a flash duration of $\left( {\frac{{ 3 \, {\text{cd} \cdot \text{s/m}}^{ 2} }}{{ 6000 \,{\text{cd/m}}^{ 2} }}} \right) = 0.5 \, {\text{ms}}$ and the constant Troland stimulus had a variable flash duration depending on the pupil size from $\left( {\frac{{32 \, {\text{Td} \cdot \text{s}}}}{{\uppi\left( {8.1 \, {\text{mm}}/2} \right)^{2} (6000 \,{\text{cd}}/{\text{m}}^{2} )}}} \right) = 0.1 \, {\text{ms}}$ to $\left( {\frac{{32 \, {\text{Td} \cdot \text{s}}}}{{\uppi\left( {1.8 \, {\text{mm}}/2} \right)^{2} ( 6000\, {\text{cd/m}}^{ 2} )}}} \right) = 2.1 \, {\text{ms}}$ for the pupil sizes measured in this study (Fig. 3). ERG timing results are measured to the center of the flash.

There was a 1 to 2 s pause (dark ganzfeld) between the first and second stimulus presented. Each stimulus was presented for at least 5 s, with the stopping criterion being the estimated standard error in the implicit time measurement being below a threshold value or 15 s, whichever comes first (141–424 flashes analyzed). Before the testing started and between the stimulus pairs, the device was removed from the patient, who sat in a normally illuminated examination room. Testing was performed with the RETeval device using sensor strip skin electrodes (LKC Technologies, Inc. Maryland, USA).

The testing order in each stimulus pair was randomly assigned by the RETeval device so that presentation order would not be a confounding variable. Because both stimuli were presented to every subject (in randomized order), the stimulus comparisons studied here should be minimally affected by differences among subjects caused by age, sex, electrode position, eye pigmentation, refractive error, and other confounding variables.

Informed consent was obtained from each subject after explanation of the nature and possible consequences of the study. The study was approved by an institutional review board (SAIRB-15-0017) and is registered at ClinicalTrials.gov (NCT02466607).

### Subjects

Subjects were enrolled at one center in the USA, half of which were dilated and half were not dilated. The inclusion criteria were healthy adult volunteers with no contraindications to pupil dilation. Exclusion criteria were pregnant women, children, subjects with light sensitivity, photosensitive epilepsy, allergies or sensitivity to pupil dilation ophthalmic solutions and history of glaucoma and cardiac dysrhythmia, as reported by potential subjects when receiving the consent form.

Subjects were tested from April to June 2015. Ages ranged from 19 to 52, with a mean age of 35. There were 8 (44%) females and 10 males.

### RETeval processing

For each of the 24 measurements, the RETeval device uses a Fourier-based approach to record the steady-state response of the retina, after excluding data near when the subject blinks (auto blink rejection as determined by a built-in infrared camera). The first 8 harmonics of the electrical response are used to reconstruct the waveform, as these harmonics contain almost all the energy [16].

**Fig. 1:**
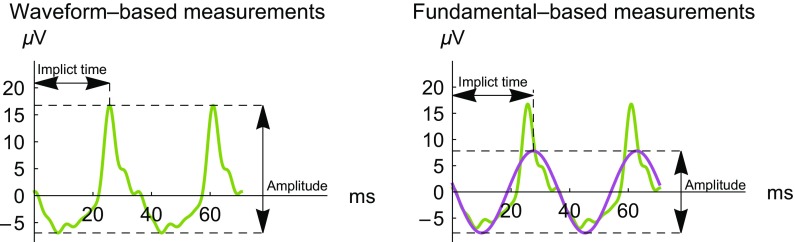
Definitions of implicit time and amplitude for the waveform (left) and fundamental (right). The fundamental of the waveform (*purple curve*) is overlaid on the original waveform (*green curve*). Time 0 is the center of the light flash; other light flashes occur at ±35.3 ms, ±70.6 ms, etc. The voltage measured is the difference between the potential at an electrode below the lower eyelid (positive) and the temple (negative) in the sensor strip electrode array

To perform a quantitative analysis of the ERG waveforms, we use two metrics for the peak-to-peak amplitude and two metrics for the implicit time (time from the center of the stimulus to the peak of the response). Figure 1 shows these definitions. The waveform-based amplitude and implicit time are measured from the response waveform, which has advantages in improved sensitivity in certain diseases [9]. The fundamental-based amplitude and implicit times use the best-fitting sinusoid, which has advantages such as robustness [16] and insensitivity to the state of light adaptation prior to the test [17].

In addition to the electrical measurements, the RETeval device also measures the pupil diameter at the flicker rate of 28.3 Hz. The IR-illuminated pupilometer camera is time-synchronized to the flicker frequency.

The RETeval device outputs a summary of results in a PDF report and extensive results in a raw data (rff) file that is suitable for automated data extraction and analysis as done here.

### Statistical methods

The pupil diameter for each step was summarized as the 20% trimmed mean of the pupil waveform, reflecting a steady-state condition.

For each subject, the slope of a linear regression fit was used to model the sensitivity of the ERG timings and amplitudes to the pupil diameter for each stimulus method. Because the amplitude of the response depends on the electrode type used, the amplitude slope is normalized to make the results transferrable to other electrodes. The amplitude slope is normalized to the percentage change from the amplitude at 2.6 mm, the average pupil diameter with natural pupils for the constant luminance stimulus. Normalization to 2.6 mm was chosen (as opposed to, for example, the average dilated pupil diameter), so that amplitude ratios would be greater than 1. The dependence of the ERG with pupil size is computed with pupil diameter because the magnitude of the residual errors was lower when fitting to the pupil diameter than when fitting to the pupil area.

Statistical analysis was done using Wolfram Mathematica^®^ 11. When comparing means (e.g., the intra-subject variability analysis), *p* values were computed with the automated testing of the function LocationEquivalenceTest, which chooses the best test type for the data: the *t* test for means when the data are normally distributed data and the Kruskal–Wallis test for medians when the data are symmetric but not normally distributed. When comparing variances (e.g., the inter-subject variability analysis), *p* values were computed with the automated testing of the function VarianceEquivalenceTest, which chooses the best test type for the data: Fisher for normal data, Levene for a robust method that is less sensitive to the normality assumption but requires symmetry around the medians, and “Brown–Forsythe” similar to Levene but does not require symmetry.

## Results

### Subjects with natural pupils

In subjects with natural pupils, the intra-subject implicit time of the fundamental and the waveform were both significantly more precise when using the constant Troland stimulus (Table 1). The precision in the amplitudes were not significantly different.

**Table 1:** Intra-subject statistics in subjects (*n* = 9) with natural pupils using constant retinal illuminance (Td) and constant luminance (cd) stimuli

	Fundamental implicit time		Fundamental amplitude			Waveform implicit time		Waveform amplitude		
	Mean/ms	SD/ms	Mean/µV	SD/µV	CV	Mean/ms	SD/ms	Mean/µV	SD/µV	CV
Td stimulus	26.1	0.26	19	1.7	8.7%,	24.9	0.25	29	2.4	8.3%
cd stimulus	27.6	0.42	17	1.4	8.3%,	25.6	0.33	24	2.0	9.0%
*P* value (test type)	0.02 (tt)	0.001 (tt)	0.4 (tt)	0.7 (tt)	0.4 (KW)	0.3 (tt)	0.01 (KW)	0.2 (tt)	0.4 (tt)	0.5 (tt)

For each subject the mean, standard deviation (SD) or cofficient of variation (CV) was computed from the 12 trials. This table shows the means across the 9 subjects for each statistics

The abbreviation “*tt*” stands for *t* test, and “*KW*” stands for Kruskal Wallis test

**Fig. 2:**
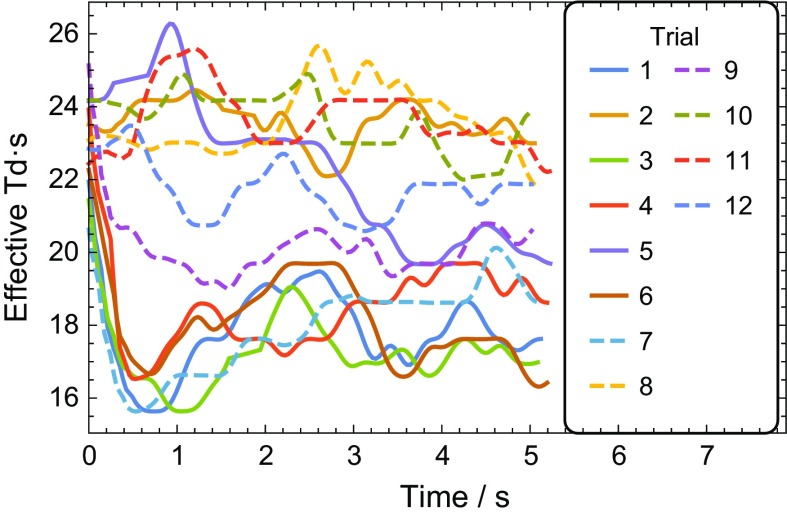
Effective retinal illuminance with a 3 cd·s/m^2^ with a 30 cd/m^2^ background, computed using the pupil measurements taken in all trials in 1 subject with natural pupils. In some trials (2, 8, 10, 11, 12), the retinal illumination was relatively constant, while in other trials (1, 3, 4, 6, 9) the retinal illumination dropped by ~1.5× in the first second, while in the remaining trails (5, 7) the retinal illumination changed slowly throughout the 5 s trial. Curves are smoothed with a 2.4 Hz low-pass filter for clarity

The pupil was measured in the constant luminance tests even though the information was not used to adjust the stimulus (Fig. 2). As shown in Fig. 2, the same stimulus does not always have the same effect on the pupil size and the pupil size sometimes changes considerably during the stimulus. The intra-subject precision of the retinal illumination in the constant luminance stimuli is computed using the standard deviation of the mean of the pupil measurements during each of the 12 trials. The intra-subject precision of the retinal illumination in the constant luminance stimuli across subjects was 11 ±  3% (mean ±  1 standard deviation). Using the 5- to 15-s-long pupil waveform for each of the 12 trials, the intra-trial precision of the retinal illumination was 7 ± 2% (mean ± 1 standard deviation). These within-run and between-run stimulus variations caused by pupil size changes may contribute to the increased variability in the constant luminance tests.

**Fig. 3:**
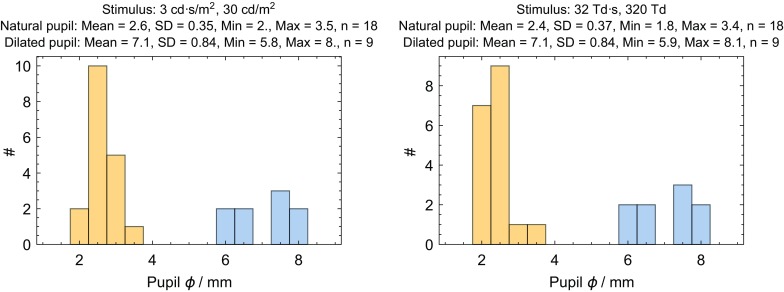
Pupil diameter (*ϕ*) distribution for the two stimuli for natural (*orange bars*) and dilated (*blue bars*) pupils. There are 18 subjects with natural pupils and only 9 subjects with dilated pupils because all subjects started with natural pupils and only half were dilated. For each group, the mean, standard deviation (SD), minimum, maximum, and number of subjects (n) are shown

The distribution of mean pupil size for the subjects with natural pupils is shown in Fig. 3. The plots include data from the first trial of all subjects. Pupil sizes ranged from 1.8 to 3.5 mm. Because these pupil sizes are smaller than the iso-luminance point between the two stimuli (3.7 mm), the Troland stimulation is brighter and the mean pupil size is smaller (2.4 vs. 2.6 mm).

Comparing the first trial from all 18 subjects (all of which had natural pupils), there were no statistically significant differences in the inter-subject variability of either implicit time or amplitude measurements (Table 2). The inter-subject variability in timing and amplitude is larger than the intra-subject variability, indicating that the device/protocol variability is dominated by variations among individuals.

**Table 2:** Inter-subject statistics in subjects (*n* = 18) with natural pupils using constant retinal illuminance (Td) and constant luminance (cd) stimuli

	Fundamental implicit time		Fundamental amplitude			Waveform implicit time		Waveform amplitude		
	Mean/ms	SD/ms	Mean/µV	SD/µV	CV	Mean/ms	SD/ms	Mean/µV	SD/µV	CV
Td stimulus	26.6	1.3	19	6.3	33%	25.1	0.92	29	8.7	31%
cd stimulus	28.2	1.7	17	6.1	36%	25.9	1.4	23	8.7	38%
*P* value (test type)	0.002 (tt)	0.3 (F)	0.3 (tt)	0.9 (F)	0.9 (F)	0.06 (tt)	0.3 (BF)	0.07 (tt)	1 (F)	1 (F)

The mean, standard deviation (SD) or cofficient of variation (CV) was computed using the first trial from all 18 subjects

The abbreviation “*tt*” stands for *t* test, “*F*” stands for Fisher Ratio or *F* test, and “*BF*” stands for Brown–Forsythe test

Temporal dependence in the ERG and pupil diameters was examined in the 9 subjects without artificial dilation. No trends or nonlinear behaviors were obvious with visual examination. Slopes from linear least squares fits were analyzed, and one (the ERG fundamental implicit time for constant retinal illumination stimulus) had a statistically significant increase of 0.25 ms across the 12 measurement time points (*p* = 0.004, where *p* = 0.005 is significant after Bonferroni correction). The remaining 7 ERG parameters and 2 pupil diameters did not have statistically significant trends.

### Subjects with artificially dilated pupils

The distribution of mean pupil size for the subjects with dilated pupils is shown in Fig. 3. The plots use data from the last trial of all dilated subjects. Pupil diameters ranged from 5.8 to 8.1 mm and were similar for both stimuli, indicating that the pupillary light reflex was effectively eliminated by the artificial mydriasis and that mydriasis was complete. Two of dilated pupil diameters were smaller than the 95% interval of a large study of dilated pupil sizes (6.4–9.2 mm) [18], although this trial used only tropicamide, while the other study [18] used both tropicamide and phenylephrine which has been shown to have a more complete dilation by about 0.6 mm [19]. The subject with smallest dilated pupil had light irides and had the smallest natural pupil of all 18 subjects in the trial. The subject’s dilated pupil size was 5.8 mm with the constant luminance stimulus and increased only 0.08 mm with the 2.4× dimmer constant retinal illuminance stimulus. The subject with the second smallest dilated pupil had dark irides and had the fourth smallest natural pupil. The subject’s dilated pupil size was 6.04 mm with the constant luminance stimulus and increased only 0.03 mm with the 2.7× dimmer constant retinal illuminance stimulus. Thus, the pupillary light reflex was effectively eliminated even in the cases that might be considered as incomplete dilators. There was not a statistically significant difference in dilated pupil size between subjects with light and dark irides (*p* = 0.06).

**Fig. 4:**
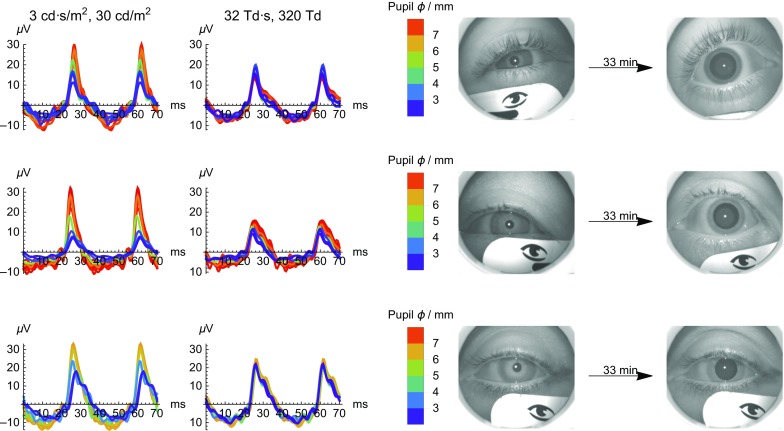
Representative ERG responses from 3 subjects (one subject per row) that received dilation drops after the first two stimulus pairs. The *left panels* show the electrical response versus time for 12 time points for the two stimuli, with the color of each curve corresponding to the measured pupil diameter (*ϕ*) as indicated by the bar chart key. The photographs on the right side are infrared photographs of the eye taken automatically by the RETeval device immediately prior to the first and last set of stimulus pairs

The constant Troland stimulation provides a more consistent retinal stimulus as the pupil changes size, as demonstrated by the greater consistency in the ERG in the first three subjects tested (Fig. 4). Most striking in the ERG waveforms is the increase in amplitude with pupil size for the constant luminance stimulation. The timing to the peak of the response also changes with the constant luminance stimulation, although the direction was inconsistent.

**Fig. 5:**
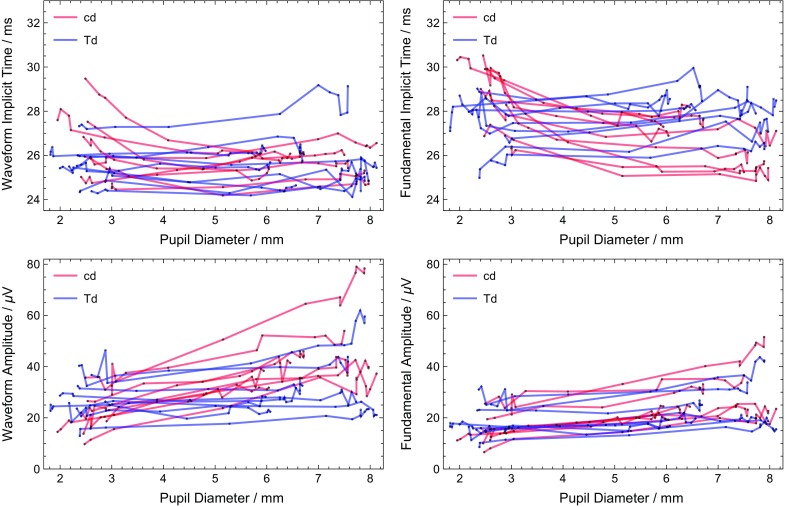
Dependence of implicit times (top plots) and amplitudes (bottom plots) on pupil size. *Red curves* are data from the constant luminance (candela) stimulus; blue curves are data from the Troland stimulus. Each subject tested has *one red* and *one blue curve*. *Black points* are the measured values, with straight lines connecting them

**Fig. 6:**
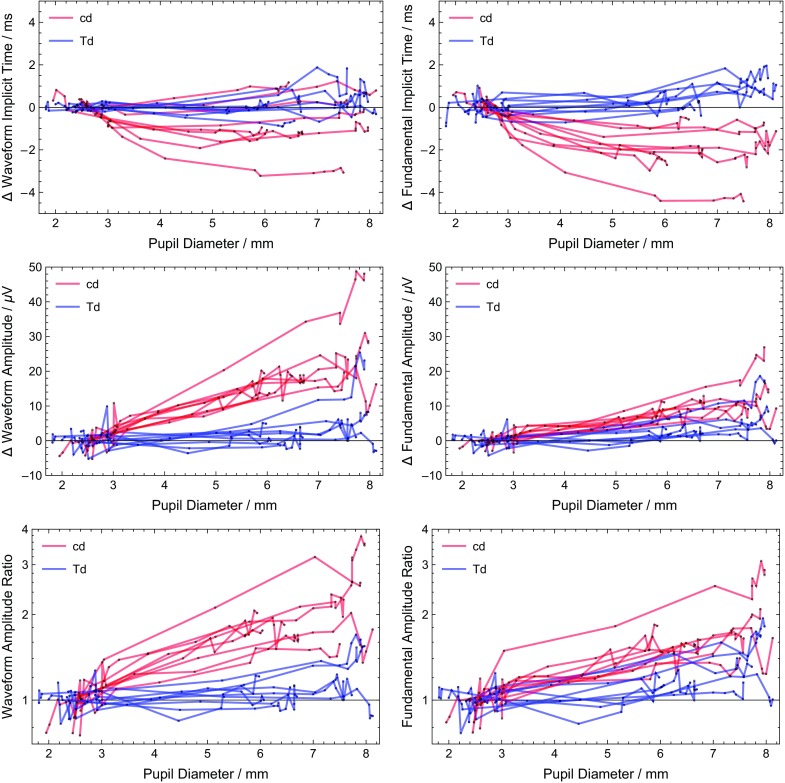
Relative dependence of implicit times (top plots) and amplitudes (middle and bottom plots) on pupil size. *Red curves* are data from the constant luminance (candela) stimulus; *blue curves* are data from the Troland stimulus. Each subject tested has *one red* and *one blue* curve. *Black points* are the measured values, with straight lines connecting them. $\Delta$ implicit times and $\Delta$ amplitudes are the difference between the measurement and the predicted measurement at a pupil size of 2.6 mm for that subject. Amplitude ratios are the ratio of the amplitude to the predicted measurement at a pupil size 2.6 mm for that subject and are plotted on a logarithmic scale. The predicted measurements are calculated using a linear least squares fit to the measurements for pupil sizes less than 5 mm. The value of 2.6 mm is the average natural pupil size found in this study

A quantitative analysis (Figs. 5 and 6) can be done using the definitions of the amplitude and implicit time for the waveform and the fundamental of the waveform (Fig. 1). Figure 5 shows the amplitude and timing for all dilated subjects. The intra-subject dependence on pupil size is difficult to see in most cases due to the inter-subject variability. Figure 6 replots the same data normalizing each subject to the average natural pupil diameter (2.6 mm) to emphasize the intra-subject pupillary dependence. The normalization was a subtraction for the implicit time and two separate methods (subtraction and ratio) for amplitudes. The amplitude ratio was analyzed in order to have results more representative across types of electrodes, as changing electrode types is primarily a scaling factor [1]. A linear least squares fit was computed for each curve, and statistics on the slopes were computed. In all 4 metrics, the Troland stimulus has less dependence of pupil size. The constant Troland stimulus tended to have a longer fundamental implicit time as function of pupil size (average slope 0.15 ms/mm, *p* = 0.003), while the constant luminance stimulus had a shorter fundamental implicit time (−0.46 ms/mm, *p* = 0.001). The waveform implicit time was essentially independent of pupil size (0.045 ms/mm, *p* = 0.3) for the Troland stimulus and the luminance stimulus (−0.13 ms/mm, * p* = 0.2), although for the luminance stimulus dependence of waveform implicit time on pupil size was highly variable among subjects. The fundamental amplitudes increased with pupil size for both the Troland stimulus (5%/mm, *p* = 0.009) and the luminance stimulus (16%/mm, *p* < 0.001). The waveform amplitude increased 22%/mm (*p* < 0.001) for the luminance stimulus and did not change for the Troland stimulus (2.7%/mm, *p* = 0.06).

By incrementally removing the largest pupil data points, the dependence of ERG parameters on pupil size becomes statistically insignificant for pupil sizes less than or equal to 6.3 mm for Troland stimulation. In other words, the Troland stimulus provided a retinal stimulation that was independent of pupil size for diameters ≤6.3 mm. In contrast, the dependence of ERG parameters on pupil size with a constant luminance stimulus was always significant.

**Fig. 7:**
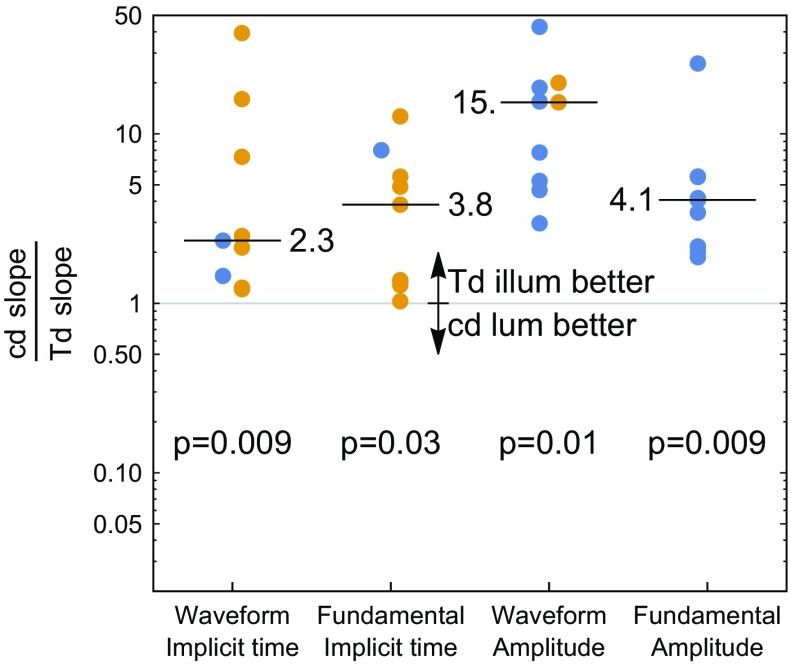
Comparison between pupil size dependence of the flicker ERG between a constant luminance (cd) and constant Troland (Td) stimulus. The ratio of the linear slope between the two stimulus conditions is shown as a *filled circle* for each subject and each waveform metric on a logarithmic scale. Positive/negative ratios are shown in *blue/gold colors*. Shown is the median line, whose value and *p* value are labeled after taking the absolute value of the points. Ratio magnitudes greater than 1 indicate that the constant retinal illuminance stimulus (Td) had less dependence on pupil size, while ratio magnitudes less than 1 indicate that the constant luminance stimulus (cd) had less dependence on pupil size

To directly compare the two stimulus conditions, the ratio between the linear least squares slopes was computed for each subject (Fig. 7). For all four quantitative waveform metrics (implicit times and amplitudes), the constant Troland stimulus had a smaller dependence on pupil size for every subject tested, as indicated by the magnitude of the slope ratio being greater than 1. The median reduction in pupil size dependence was a factor of 2.3, 3.8, 4.1, and 15 depending on which waveform metric is considered.

Table 3 compares the inter-subject variability between a natural pupil Troland stimulus and the ISCEV standard dilated pupil constant luminance stimulus. The Troland analysis uses the first measurement for each dilated subject (before dilation drops were instilled), while the luminance analysis uses the last measurement for each dilated subject (30 min after instillation). There are no statistically significant differences in inter-subject variability between these two conditions for any of the four quantitative measures.

**Table 3:** Inter-subject statistics (*n* = 9) between of a natural pupil Troland stimulus and a dilated constant luminance stimulus

	Fundamental implicit time		Fundamental amplitude			Waveform implicit time		Waveform amplitude		
	Mean/ms	SD/ms	Mean/µV	SD/µV	CV	Mean/ms	SD/ms	Mean/µV	SD/µV	CV
Td stimulus	27.3	1.3	18	7.1	39%	25.5	0.93	27	8.7	32%
cd stimulus	26.5	1.1	26	10	40%	25.7	0.49	42	15	36%
*P* value (test type)	0.2 (tt)	0.9 (L)	0.08 (tt)	0.9 (BF)	0.6 (BF)	0.5 (tt)	0.2 (L)	0.02 (tt)	0.7 (F)	0.1 (F)

The standard deviation (SD) or cofficient of variation (CV) was computed across the 9 subjects

The abbreviation “*tt*” stands for *t* test, “*F*” stands for Fisher Ratio or *F*-test, “*L*” stands for Levene test and “BF” stands for Brown–Forsy the test

## Discussion

This study compared the flicker ERG from two different stimuli—a constant luminance stimulus and a constant Troland stimulus scaled to provide the same retinal illumination with a 3.7 mm pupil.

Using the data from the dilated subjects to determine the dependence of ERG parameters on pupil size, we found that the Troland stimulus elicits flicker ERGs that are independent of pupil size for pupils smaller than 6.3 mm. These results are consistent with that of Kato et al. [15], who found that the Troland stimulus provided a pupil-independent stimulus for pupil diameters up to 6.5 mm. For larger pupil diameters, the Stiles–Crawford effect [20, 21] likely causes a constant Troland stimulus to provide less effective retinal stimulation, as the larger pupil annuli are less sensitive to light than would be expected based solely on pupil geometry. The less effective retinal stimulation hypothesis is consistent with the Troland stimulation’s residual dependence on implicit time (longer with increasing pupil size), but is inconsistent with amplitude (greater amplitude with increasing pupil size).

The pupillary dependence of the ERG using the constant luminance stimulus may result from changes in the retinal illumination of the flickering light or the background light. For example, one study [6] reported a 3.3 ms faster 30 Hz flicker implicit time (from 28.0 to 24.7 ms) when a background light was turned on.

The constant luminance results in Figs. 5 and 6 can be examined for the presence of a photopic hill [22], which has been reported in single flash photopic recordings to have a maximal response near 3 cd·s/m^2^ with a 30 cd/m^2^ background [23]. No such curvature is noted in this study, although the stimuli used are likely too dim to see an effect. Unpublished work by the authors did not see a downward trend in the flicker ERG amplitude in two subjects with stimuli as bright as 400 Td·s.

During the 3 min between trials, the ganzfeld was removed from the subject who sat in a normal examination room lighting. Since this office lighting is not as controlled as the ganzfeld, there was a chance that the eye’s adaptation state may change throughout the experiment. The data indicate that this possibility was not an issue because the subjects not dilated did not show a trend over time in the ERG and pupil size for 9 of the 10 metrics examined.

The intra-subject variability in implicit time was smaller with the Troland stimulus, and the Troland stimulus had less dependence on pupil size. These results are consistent with the variability in ERG results being due in part to pupil size changes that occur in short time frames. One alternative to continuously monitoring and adjusting for pupil size is to measure and compensate for pupil size once at the beginning of the test in order to reduce the ERG’s dependency on pupil size. Figure 2 shows that this approach would not eliminate variability caused by pupil size because natural pupil change size throughout the stimulus duration. A quantitative analysis of this strategy can be estimated (although inexactly because the stimulus was not adjusted once in this study) by looking at the pupil waveforms for the 12 trials for the 9 control subjects when stimulated with constant luminance stimulus. The retinal illuminance energy for those 108 conditions varied from 48 Td·s to 7 Td·s—a range of 41 Td·s, with a mean retinal illuminance energy of 16 Td·s. Adjusting once immediately after the stimulus starts reduces that range to 21 Td·s (in Fig. 2, subject had a range of 9 Td·s, with a mean of 12 Td·s). Adjusting once 1 s after the stimulus starts has a range of 13 Td·s (in Fig. 2, subject was 4 Td·s). Thus, an adjust-once strategy, while being 2–3 times better than never adjusting the luminance, is still expected to have significant changes in retinal illumination (stimulus range is 81–130% of the mean).

We expect that natural variations in pupil size would contribute to the inter-subject variability; however, the natural pupil size range in our subjects (1.8–3.5 mm) did not cause a measurable increase in inter-subject variability when comparing a constant luminance stimulus with a Troland stimulus. Nevertheless, changes in ERG parameters associated with pupil size are expected to increase reference range sizes. As shown in Fig. 6, the waveform amplitude dependence on pupil diameter is fairly linear with the constant luminance stimulus. From a study of 250 subjects (500 eyes) [24], the 95% interval of natural pupil sizes in direct light is about 3.1 mm (1.7–4.8 mm), leading to, for example, a waveform amplitude change of 68% (= (3.1 mm)(22%/mm)) due to variation in pupil size. For dilated pupils, a different study of 484 subjects [18] found the 95% interval of dilated pupil diameters to be 2.8 mm (6.4–9.2 mm). The measured pupillary dependence predicts a waveform amplitude change of 62% (= (2.8 mm)(22%/mm)) due to variation in pupil size in dilated pupils. Thus, pupil size may be a major contributor to the size of reference ranges in ERG tests. These data suggest that reference ranges may be smaller with constant Troland stimuli with a corresponding improvement in the sensitivity for detecting abnormality or change in flicker ERG parameters.

As described in the ISCEV standard [1], ERG tests are most often performed with dilated pupils. Pupil dilation is inconvenient for the patient, but it reduces the variability in constant luminance tests caused by pupil size, as the pupil size range is somewhat smaller when dilated. Nevertheless, Table 3 demonstrates that the inter-subject variability is equivalent between a natural pupil Troland test and a dilated constant luminance test. Thus, a Troland test minimizes residuals risks associated with artificial mydriasis, which includes acute closed-angle glaucoma [25] and impaired driving [26], while providing equivalent inter-subject variability.

A limitation of this study is we measured the flicker ERG with only one Troland stimulus, and therefore, any dependencies of ERG parameters on retinal illumination using a Troland stimulus could not be explored. Based on a typical Stiles–Crawford coefficient of 0.05 [21] and the 95% reference range of dilated pupil diameters from above, the effective retinal illumination from the ISCEV standard stimulus with a dilated pupil is between 76 Td·s and 123 Td·s with a background between 760 Td and 1230 Td). The selected Troland stimulus (32 Td·s with a 320 Td background) was chosen to balance the retinal illumination to be sometimes brighter and sometimes dimmer as the pupil diameter changed with the constant luminance stimulus in order to best provide equivalent stimuli for pupillary dependence measurements. However, this selected Troland stimulus is 2.4–3.8 times dimmer than an ISCEV equivalent stimulus, making the variability comparison between dilated ISCEV stimulus and the natural pupil Troland stimulus imperfect. However, as shown in Figs. 5 and 6, brighter flicker stimuli elicit larger amplitudes and therefore likely have less variable ERG responses due to a smaller relative contribution of electronic noise; therefore, a brighter Troland stimulus may compare even more favorably to the ISCEV standard luminance with dilated pupils.

Another limitation of this study is we only measured the flicker ERG, which a cone-driven response [27]. Rods play an important role in dark-adapted ERG tests, which do not have a Stiles–Crawford effect [21, 28]. Therefore, we expect that Troland-based stimulation would have a smaller dependence on pupil size, although this is yet to be proven by experimental results.

In conclusion, we found that the dependence on pupil size of the flicker ERG for Troland-based stimuli to be not measurable below a pupil size of 6.3 mm. Further, we found the inter-subject variability for non-dilated Troland stimuli to be equivalent to the dilated constant luminance stimuli. Therefore, the use of constant retinal illuminance (Troland stimulus) with natural pupils for flicker ERGs provides more efficient testing with the potential for increased sensitivity.

## References

[1] McCulloch DL, Marmor MF, Brigell MG, Hamilton R, Holder GE, Tzekov R, Bach M (2015). ISCEV Standard for full-field clinical electroretinography (2015 update). Doc Ophthalmol Adv ophthalmol.

[2] Massof RW, Johnson MA, Sunness JS, Perry C, Finkelstein D (1986). Flicker electroretinogram in retinitis pigmentosa. Doc Ophthalmol Adv Ophthalmol.

[3] Berson EL (1993). Retinitis pigmentosa. The friedenwald lecture. Invest Ophthalmol Visual Sci.

[4] Hood DC, Birch DG (1996). Abnormalities of the retinal cone system in retinitis pigmentosa. Vision Res.

[5] Sieving PA, Arnold EB, Jamison J, Liepa A, Coats C (1998). Submicrovolt flicker electroretinogram: cycle-by-cycle recording of multiple harmonics with statistical estimation of measurement uncertainty. Invest Ophthalmol Visual Sci.

[6] Bresnick GH, Palta M (1987). Temporal aspects of the electroretinogram in diabetic retinopathy. Arch Ophthalmol.

[7] Holopigian K, Seiple W, Lorenzo M, Carr R (1992). A comparison of photopic and scotopic electroretinographic changes in early diabetic retinopathy. Invest Ophthalmol Visual Sci.

[8] Tahara K, Matsuura T, Otori T (1993). Diagnostic evaluation of diabetic retinopathy by 30-Hz flicker electroretinography. Jpn J Ophthalmol.

[9] Maa AY, Feuer WJ, Davis CQ, Pillow EK, Brown TD, Caywood RM, Chasan JE, Fransen SR (2016). A novel device for accurate and efficient testing for vision-threatening diabetic retinopathy. J Diabetes Complications.

[10] Severns ML, Johnson MA (1993). Predicting outcome in central retinal vein occlusion using the flicker electroretinogram. Arch Ophthalmol.

[11] Larsson J, Andreasson S (2001). Photopic 30 Hz flicker ERG as a predictor for rubeosis in central retinal vein occlusion. Br J Ophthalmol.

[12] Yasuda S, Kachi S, Kondo M, Ushida H, Uetani R, Terui T, Piao CH, Terasaki H (2011). Significant correlation between electroretinogram parameters and ocular vascular endothelial growth factor concentration in central retinal vein occlusion eyes. Invest Ophthalmol Visual Sci.

[13] Yasuda S, Kachi S, Ueno S, Piao CH, Terasaki H (2015). Flicker electroretinograms before and after intravitreal ranibizumab injection in eyes with central retinal vein occlusion. Acta Ophthalmol.

[14] Westall CA, Wright T, Cortese F, Kumarappah A, Snead OC, Buncic JR (2014). Vigabatrin retinal toxicity in children with infantile spasms: an observational cohort study. Neurology.

[15] Kato K, Kondo M, Sugimoto M, Ikesugi K, Matsubara H (2015). Effect of pupil size on flicker ERGs recorded with RETeval system: new mydriasis-free full-field ERG system. Invest Ophthalmol Visual Sci.

[16] Severns ML, Johnson MA, Merritt SA (1991). Automated estimation of implicit time and amplitude from the flicker electroretinogram. Appl Opt.

[17] McAnany JJ, Nolan PR (2014). Changes in the harmonic components of the flicker electroretinogram during light adaptation. Doc Ophthalmol Adv Ophthalmol.

[18] Hammond CJ, Snieder H, Spector TD, Gilbert CE (2000). Factors affecting pupil size after dilatation: the Twin eye study. Br J Ophthalmol.

[19] Huber MJ, Smith SA, Smith SE (1985). Mydriatic drugs for diabetic patients. Br J Ophthalmol.

[20] Stiles WS, Crawford BH (1933). The luminous efficiency of rays entering the eye pupil at different points. Proc R Society of Lond Ser B.

[21] Westheimer G (2008). Directional sensitivity of the retina: 75 years of stiles-crawford effect. Proc Biol Sci.

[22] Wali N, Leguire LE (1992). The photopic hill: a new phenomenon of the light adapted electroretinogram. Doc Ophthalmol Adv Ophthalmol.

[23] Rufiange M, Dassa J, Dembinska O, Koenekoop RK, Little JM, Polomeno RC, Dumont M, Chemtob S, Lachapelle P (2003). The photopic ERG luminance-response function (photopic hill): method of analysis and clinical application. Vis Res.

[24] Richman JE, McAndrew KG, Decker D, Mullaney SC (2004). An evaluation of pupil size standards used by police officers for detecting drug impairment. Optometry.

[25] Liew G, Mitchell P, Wang JJ, Wong TY (2006). Fundoscopy: to dilate or not to dilate?. BMJ.

[26] Wood JM, Garth D, Grounds G, McKay P, Mulvahil A (2003). Pupil dilatation does affect some aspects of daytime driving performance. Br J Ophthalmol.

[27] Kondo M, Sieving PA (2002). Post-photoreceptoral activity dominates primate photopic 32-Hz ERG for sine-, square-, and pulsed stimuli. Invest Ophthalmol Visual Sci.

[28] Flamant F, Stiles WS (1948). The directional and spectral sensitivities of the retinal rods to adapting fields of different wave-lengths. J Physiol.

